# *Fusobacterium nucleatum* induces MDSCs enrichment *via* activation the NLRP3 inflammosome in ESCC cells, leading to cisplatin resistance

**DOI:** 10.1080/07853890.2022.2061045

**Published:** 2022-04-18

**Authors:** Mengxia Liang, Yiwen Liu, Zheyuan Zhang, Haijun Yang, Ningtao Dai, Ning Zhang, Wei Sun, Yibo Guo, Jinyu Kong, Xiaopeng Wang, Min Wang, Fuyou Zhou

**Affiliations:** a Anyang Tumor Hospital, Anyang Tumor Hospital affiliated to Henan University of Science and Technology, Henan Key Medical Laboratory of Precise Prevention and Treatment of Esophageal Cancer, Anyang, China; b Henan Key Laboratory of Microbiome and Esophageal Cancer Prevention and Treatment; Henan Key Laboratory of Cancer Epigenetics; Cancer Hospital, The First Affiliated Hospital（College of Clinical Medicine）of Henan University of Science and Technology, Luoyang, China; c The Third Affiliated Hospital of Xinxiang Medical University, Xinxiang, China

**Keywords:** Oesophageal squamous cell carcinoma, *Fusobacterium nucleatum*, inflammasome, myeloid-derived suppressor cells, cisplatin

## Abstract

**Background:**

To analyse the regulatory effect of *Fusobacterium nucleatum* (Fn) on NOD-like receptor protein 3 (NLRP3) and myeloid-derived suppressor cells (MDSCs) in oesophageal squamous cell carcinoma (ESCC) as well as its effect on cisplatin (CDDP) therapy and to explore its clinical significance.

**Methods:**

Fn infection, NLRP3 expression and MDSCs infiltration in ESCC tissues were detected by RNAscope and immunohistochemistry (IHC). The correlation between these three factors and the clinicopathological features and survival of ESCC patients was analysed. A coculture system of human peripheral blood monocytes (PBMCs) and ESCC cells was established to simulate the tumour microenvironment. *In vitro* and *in vivo* models were used to analyse the effects of Fn on the percentage of MDSCs in the coculture system and the NLRP3 expression level and CDDP sensitivity of ESCC cells.

**Results:**

Fn infection was consistent with high NLRP3 expression and MDSCs enrichment in ESCC tissues. Moreover, the survival time of ESCC patients was significantly shortened under Fn infection, high NLRP3 expression and MDSCs enrichment. In the *in vitro* and *in vivo* models, Fn induced abundant enrichment of MDSCs by inducing high expression of NLRP3 in ESCC cells and reducing the sensitivity of ESCC cells to CDDP.

**Conclusions:**

Fn infection can induce high expression of NLRP3 in ESCC, lead to MDSCs enrichment, weaken the body's antitumour immunity, and lead to CDDP treatment resistance. The effective elimination of Fn and the inhibition of MDSCs enrichment may provide new strategies and treatments for ESCC.

**Highlights**
- The survival of ESCC patients with Fn infection, high NLRP3 expression and MDSCs enrichment was significantly shortened.
- Fn infection could cause CDDP resistance in ESCC.
- Fn could induce the enrichment of MDSCs in the tumour microenvironment by activating NLRP3 in ESCC cells.

## Background

The incidence and mortality of oesophageal cancer are extremely high. More than 90% of oesophageal cancer cases are oesophageal squamous cell carcinoma (ESCC), and most patients have moderate or advanced disease at diagnosis [1]. One of the most important treatments for patients with advanced ESCC is chemotherapy. Cisplatin (CDDP) is a commonly used chemotherapy drug that exhibits a good response but easily produces drug resistance [2]. Therefore, it is of great significance to identify the molecular mechanism of CDDP resistance in ESCC to improve treatment methods and their clinical efficacy. Previous studies by our team have confirmed that *Fusobacterium nucleatum* (Fn) infection is closely related to the occurrence and development of ESCC, but its specific pathogenic mechanism is not completely clear [3–4]. Various pathogenic microorganisms can regulate the host immune response by triggering inflammatory responses, assist tumour cells in escaping immune surveillance, and provide favourable conditions for their long-term colonization [5]. NOD-like receptor protein 3 (NLRP3) in the inflammasome is a key inducer of the inflammatory response [6]. It can regulate immune cells to prevent their normal functions, reshape the immune microenvironment, promote the malignant progression of a variety of tumours and assist in the development of chemotherapy resistance [7–8]. Myeloid-derived suppressor cells (MDSCs) are a type of undifferentiated and mature HLA-DR^−^CD11b^+^CD33^+^ cells [9–11] with immunosuppressive functions that play an important role in maintaining the body’s tolerance and regulating the immune response level, and they can mediate the immune escape of tumour cells, which is the main obstacle in tumour immunotherapy [12–13]. Clinical data suggest that the overactivation of NLRP3 is closely related to the enrichment of MDSCs in a variety of tumours [14–15].

In this study, Fn infection, NLRP3 expression and MDSCs infiltration in ESCC tissues were first detected, and their correlations with clinicopathological features and survival were analysed. Then, a coculture system of human peripheral blood mononuclear cells (PBMCs) and ESCC cells was established to preliminarily explore the regulatory effects of Fn on NLRP3 and MDSCs in ESCC and the associated effects on CDDP therapy. Finally, *in vitro* and *in vivo* experiments were conducted to study the overall molecular mechanism by which Fn can activate NLRP3 in ESCC cells to cause the massive enrichment of MDSCs and lead to CDDP treatment resistance. The results provide new insights into the treatment of ESCC.

## 1. Materials and methods

### 1.1. Cells, tissues, and subjects

**Figure 1.**
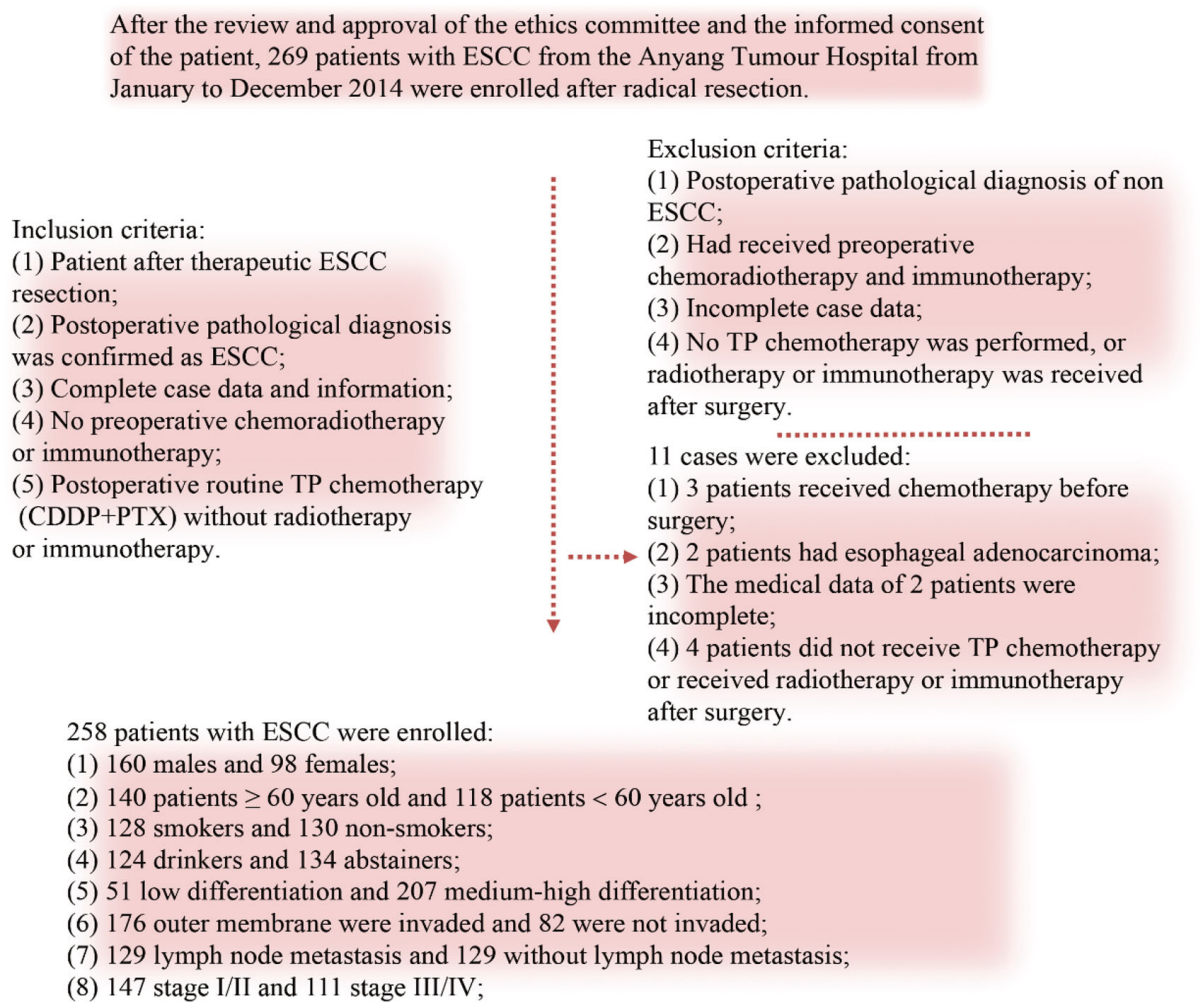
Enrolment criteria.

The human ESCC KYSE150 cell line and 293 T cell line (stored frozen in the laboratory) as well as the Fn strain ATCC 2558 (donated by The University of Louisville School of Stomatology) were used for this study. A total of 258 patients with ESCC were ultimately enrolled in the study. The inclusion criteria for the patients are shown in Figure 1 (ethics code: 2021WZ10K01).

### 1.2. RNAscope

Conventional paraffin-embedded ESCC tissue sections (2.5 μm thick) were dewaxed before treatment with hydrogen peroxide, antigen retrieval buffer (China Solarbio Co.) and Protease Plus, after which they were hybridised with the Fn probe (ACD Co. of America) at 40 °C for 2 h. Amp 1-Amp 6 and RED working solution (ACD Co. of America) were successively added to the samples and incubated, and then the sections were stained with haematoxylin. The slides were mounted with a sealing tablet. Red puncta in the ESCC tissue sections indicated Fn infection (16S rRNA). The determination of positive cells and positive samples was based on references [16] and [17]. In this study, the criteria for Fn infection positivity were as follows: ① ≥ 5 single red particles in a cell and ② > 30% of positive cells per section [16–17].

### 1.3. Immunohistochemical analysis

Three conventional paraffin-embedded ESCC tissue sections (2.5 μm thick) were collected from each patient. After dewaxing, hydration, antigen retrieval, peroxidase blocking (Beijing Zhongshan Jinqiao Biotechnology Co.) and goat serum blocking, NLRP3, CD11b and CD33 (Abcam Co. of UK, 1 : 200 diluted with phosphate-buffered saline (PBS)), antibodies were added to the samples for incubation. After cleaning with PBS, the cells were incubated with goat anti-rat/rabbit polymer (Beijing Zhongshan Jinqiao Biotechnology Co.), sealed with SP (Beijing Zhongshan Jinqiao Biotechnology Co.), stained with DAB (China Solarbio Co.), redyed with haematoxylin, dehydrated with a gradient series of ethanol, and sealed with neutral gum after clearing with xylene [18–19]. The results were evaluated as follows. ESCC cells with brownish yellow or brown granules in the cytoplasm were positive for NLRP3 expression. The presence of brownish yellow or brown particles on the membrane of monocytes at the same location in two consecutive sections of ESCC tissue indicated positive MDSCs infiltration (CD11b and CD33 were both positive). The positive control, negative control and immunohistochemical scoring criteria are listed in references [20] and [21]. In this study, the criteria used for NLRP3 expression were as follows: 0 was defined as negative, and ≥1 was defined as positive [20]. MDSCs infiltration was reported as follows: a score of 0 was defined as negative, and a score ≥1 was defined as positive [21].

### 1.4. Lentivirus packaging

293T cells were cultured in a 5% CO_2_ incubator at 37 °C. Transfection began when the confluency of the 293 T cells reached 60% ∼ 70%. An NLRP3–DNA–EndoFectin mixture (GeneCopeia Co. of America) was prepared and added to the 293 T cells. After conventional culturing for 12 h, the medium was replaced with fresh medium supplemented with “Titer Boost” (1/500 volume) (GeneCopeia Co. of America). After 48 h of routine culturing, the supernatant containing viral particles was collected and filtered through a 0.45-μm filter to remove cell debris. When the KYSE150 cells reached 50% ∼ 60% confluency, the virus-containing supernatant was added to the cells, which were then cultured in the presence of puromycin for 2 weeks to screen for infected cells. Western blot was used to detect the knockdown efficiency of NLRP3. Cells with successful NLRP3 knockdown were named KYSE150-N.

### 1.5. Establishment of a coculture system

Peripheral blood from healthy individuals was collected in an anticoagulant tube and diluted 1:1 with normal saline. An appropriate amount of Ficoll-Paque separation solution (GE Co. of America) was added to the blood before it was centrifuged at 800 ×*g* for 20 min. After separation, the white film layer was carefully collected and centrifuged at 800× *g* for 5 min. The pellet was washed twice with PBS and resuspended to produce a PBMC solution. The PBMC cell count was adjusted to 1 × 10^6^ cells/mL in RPMI 1640 medium (containing 10% foetal bovine serum). The remaining cells were treated with 1000 U/mL IFN-γ (PeproTech Co. of America) and cultured at 37 °C in a 5% CO_2_ incubator for 24 h. Then, 50 ng/mL OKT3 (PeproTech Co. of America), 100 U/mL IL-1β (PeproTech Co. of America) and 1000 U/mL IL-2 (PeproTech Co. of America) were added. The medium was replaced every 3 days. KYSE150 and KYSE150-N cells were routinely cultured in RPMI 1640 medium (Gibco Co. of America) containing 10% foetal bovine serum (Gibco Co. of America). When the cells reached 60% ∼ 70% confluence, PBMCs were added to the cell culture medium at a ratio of 10:1 and coincubated at 37 °C in a 5% CO_2_ incubator.

### 1.6. Fn infection of ESCC cell cultures

Fn strain ATCC 2558 was cultured in brain-heart infusion medium (Merieux Alliance of French) containing 5% sterile cotton sheep blood (China Solarbio Co.), 1% haem chloride (China Solarbio Co.) and 0.1% vitamin K1 (China Solarbio Co.) in an anaerobic workstation (COY Co. of America) containing 85% N_2_, 10% H_2_ and 5% CO_2_ at 37 °C. The Fn purity was determined by Gram staining on Columbia blood agar plates. The Fn bacterial solution with the best vitality was selected (OD600 = 1∼2). When the cell density was 60% ∼ 70%, Fn was added to the cell culture medium at multiplicity of infection (MOI)=10. The infection time was 72 h. Cells infected with Fn were named KYSE150 + Fn and KYSE150-N + Fn.

### 1.7. Western blot

After processing fresh cell or tissue samples, the protein concentration was detected and quantified with a BCA protein quantification kit (China Kangwei Biological Technology Co.). The sample size per lane was 30 µg total protein. The proteins were separated by SDS–PAGE with a 10% mass fraction and transferred to PVDF membranes (Millipore Co., America). TBST containing 5% skim milk maintained at room temperature for 1 h was used to block the membrane. NLRP3 and GAPDH (Abcam Co. of UK, 1:2000 TBST dilution) antibodies were added and incubated at 4 °C overnight. After rewarming, secondary antibody (China Kangwei Biological Technology Co., 1:2000 TBST dilution) was added and incubated for 2 h. An ECL luminescent developer (Invitrogen Co. of America) was used to detect the target protein bands, a gel imaging system (BIO-RAD Co. of America) was used to collect images, and Image Lab software was used to measure the grey value of the protein bands. The grey value ratio between the target protein and the reference protein bands was used as the final relative protein expression [22–23]. The experiment was repeated three times.

### 1.8. Flow cytometry

MDSCs were stained with anti-CD45/APC-CY7 (Biolegend Co. of America), anti-HLA-DR/FITC (Biolegend Co. of America), anti-CD11b/APC (Biolegend Co. of America) and anti-CD33/PE (Biolegend Co. of America) fluorescently labelled antibodies. The percentage of MDSCs was detected by flow cytometry (Beckman Coulter Co. of America). Leukocytes (CD45^+^ cells) were separated from monocytes by morphological gating based on side scattering (SSC) and forward scattering (FSC) values. The HLA-DR cell population was also separated from the leukocyte population. Finally, CD11b^+^CD33^+^ MDSCs were selected from the CD45^+^HLA-DR^−^ cell population. Representative flow cytometry of each group was used to identify the leukocyte, HLA-DR^−^ cell and MDSCs (CD45^+^HLA-DR^−^CD11b^+^CD33^+^ cells) populations. The data were analysed using Summit 5.3 software, and the results are expressed as the percentage of positive cells [24]. The experiment was repeated three times.

### 1.9. CCK8

The CCK-8 method was used to detect the half maximal inhibitory concentration (IC50) of CDDP. Cells in each group were treated in advance (for example, the time of Fn infection in the 24 h group, 48 h group and 72 h group from the beginning to the end were 24 h, 48 h and 72 h, respectively). A 100-μl cell suspension was prepared in a 96-well plate (with 1000 cells in each well) and precultured in an incubator for 24 h (37 °C, 5% CO_2_). The control group and experimental groups were treated with different concentrations (0.25, 0.5, 0.75, 1.0, …, 25.0 μg/mL) of CDDP (Selleck Chemicals Co. of America). CDDP was added to the groups according to the indicated concentration, and the plates were cultured for 24 h. Finally, CCK-8 reagent (Dojindo China Co.) was added to each plate (10 μl/well). After 1 h, the medium was replaced with fresh medium, and the absorbance at 450 nm was measured with a microplate reader. The 24 h median inhibitory concentration of CDDP (IC50) in each group was calculated, and the IC50 values of the different groups were compared [25]. The experiment was repeated three times.

### 1.10. *In vivo* tumour studies

Twenty-four healthy 4-week-old female NSG mice weighing approximately 20 g were housed under specific pathogen-free (SPF) conditions at a temperature of 25 ± 2 °C and a humidity of 40–60% with natural circadian lighting. The NSG mice were provided SPF feed and drinking water ad libitum. After 1 week of adaptive feeding, they were randomly divided into four groups: KYSE150 + Fn, KYSE150-N + Fn, KYSE150 + Fn + CDDP and KYSE150-N + Fn + CDDP (with 6 mice in each group raised in SPF cages). KYSE150 + Fn and KYSE150-N + Fn cells were labelled with PKH67 (Sigma Co. of America) fluorescent dye. All cells showed fluorescence. A total of 1 × 10^6^ cells were inoculated into the right axilla of each NSG mouse. After the tumours of the NSG mice in each group grew to approximately 0.5–1 cm in diameter, approximately 1 × 10^7^ human PBMCs were injected into the mice in each group *via* the tail vein. Meanwhile, each CDDP (Selleck Chemicals Co. of America) treatment group was injected with 5 mg/kg CDDP every 3 days *via* the tail vein [26]. Tumour volumes were measured with a Vernier calliper during the experiment. The tumour size was detected by using a small animal imager (PerkinElmer Co. of America). Finally, the NSG mice were sacrificed by cervical dislocation, and the tumour bodies were excised and weighed. The tumours were ground after weighing. After selected proteins were extracted, the expression of the NLRP3 protein in the NSG mice was detected by western blot. The remaining samples were prepared as single-cell suspensions, and the percentage of MDSCs in the NSG mice in each group was detected by flow cytometry. The animal study was carried out in accordance with the Anyang Tumour Hospital animal care guidelines (ethics code: 2021WZ10K01).

### 1.11. Statistical treatment

Statistical analysis was performed using SPSS 26.0 software. Measurement data are expressed as x̄±s, and univariate ANOVA was used to compare differences among groups. Statistical data were analysed by the chi-square test. The Kaplan–Meier method was used to draw the survival curves. Differences in survival were analysed by the log-rank method. The survival time was defined as the time from admission to the date of the last follow-up or death. The length of the follow-up was 60 months. The deleted data were associated with patients who were still alive after 60 months of follow-up, while the undeleted data were associated with patients who died due to ESCC. *p* < 0.05 indicated a significant difference.

## 2. Results

### 2.1. Detection of Fn infection, NLRP3 expression and MDSCs enrichment in ESCC tissues

**Figure 2.**
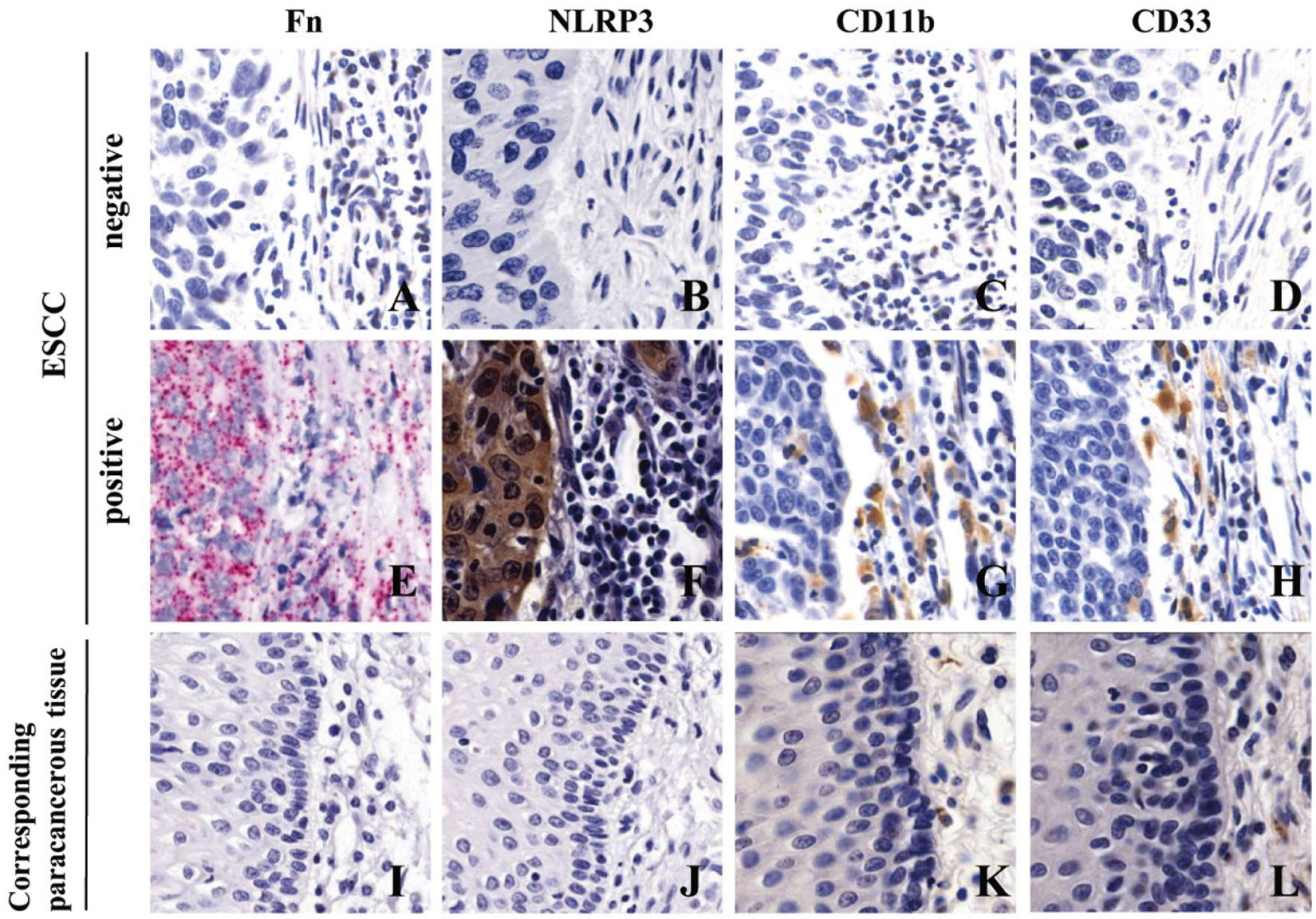
Fn infection, NLRP3 expression and MDSCs enrichment in ESCC tissues (×400). A/E/I: Fn infection was detected by RNAscope (16S rRNA); B/F/J: NLRP3 expression was detected by IHC; C/D/G/H/K/L: MDSCs infiltration (CD11b and CD33) was detected by IHC.

We used RNAscope and IHC to detect Fn infection, NLRP3 expression and MDSCs infiltration in cancer tissues and adjacent tissues collected from 258 ESCC patients treated with conventional TP chemotherapy after surgery. Diffuse red particles were found in the cytoplasm of cancer cells and stromal cells, indicating positive Fn infection (Figure 2(E)), while adjacent tissues were mostly negative for Fn infection (*p* < 0.05, Figure 2(I) and Table 1). Brownish yellow granules were present in the cytoplasm of cancer cells at the same position in sequential sections, indicating positive NLRP3 expression (Figure 2(F)), while was the corresponding adjacent tissues were mostly negative for NLRP3 expression (*p* < 0.05, Figure 2(J) and Table 1). In addition, brownish yellow particles were present on the membranes of monocytes at the same location in consecutive sections, indicating positive MDSCs enrichment (Figure 2(G and H), CD11b^+^CD33^+^), while less MDSCs infiltration was exhibited in corresponding adjacent tissues (*p* < 0.05, Figure 2(K, L) and Table 1). Meanwhile, there was significant consistency among Fn infection, high NLRP3 expression and MDSCs enrichment in ESCC tissues (Figure 2 and Table 2). In conclusion, NLRP3 was highly expressed and MDSCs were enriched in ESCC tissues with Fn infection. In contrast, ESCC tissues without Fn infection showed low NLRP3 expression and less MDSCs infiltration.

**Table 1.** Correlation analysis of various factors in ESCC cancer tissues and adjacent tissues.

	Oesophageal squamous cell carcinoma tissue					
	Fn(+)	Fn (-)	NLRP3(+)	NLRP3(-)	MDSCs(+)	MDSCs(-)
Corresponding paracancerous tissue						
Fn (+)	9 (3.49)^a^	0 (0.00)^a^	–	–	–	–
Fn (-)	76 (29.46)^a^	173 (67.05)^a^	–	–	–	–
NLRP3(+)	–	–	20 (7.75)^b^	0 (0.00)^b^	–	–
NLRP3(-)	–	–	98 (37.99)^b^	140 (54.26)^b^	–	–
MDSCs(+)	–	–	–	–	8 (3.10)^c^	0 (0.00)^c^
MDSCs(-)	–	–	–	–	71 (27.52)^c^	179 (69.38)^c^

^a^Chi-square test comparing the positive rates of Fn infection in cancer tissues and adjacent tissues, *p* < 0.05.

^b^Chi-square test comparing the positive rates of NLRP3 overexpression in cancer tissues and adjacent tissues, *p* < 0.05.

^c^Chi-square test comparing the positive rates of MDSCs enrichment in cancer tissues and adjacent tissues, *p* < 0.05.

**Table 2.** Consistency analysis of various factors in ESCC tissues.

		Fn		NLRP3		MDSCs	
		(+)	(-)	(+)	(-)	(+)	(-)
Fn	(+)	–	–	78 (30.23)^b^	7 (2.72)^b^	77 (29.84)^c^	8 (3.10)^c^
	(-)	–	–	40 (15.50)^b^	133 (51.55)^b^	2 (0.78)^c^	171 (66.28)^c^
NLRP3	(+)	78 (30.23)^a^	40 (15.50)^a^	–	–	75 (29.07)^c^	43 (16.67)^c^
	(-)	7 (2.72)^a^	133 (51.55)^a^	–	–	4 (1.55)^c^	136 (52.71)^c^
MDSCs	(+)	77 (29.84)^a^	2 (0.78)^a^	75 (29.07)^b^	4 (1.55)^b^	–	–
	(-)	8 (3.10)^a^	171 (66.28)^a^	43 (16.67)^b^	136 (52.71)^b^	–	–

^a^Cohen's kappa coefficient was used to analyse the consistency with Fn infection, *p* < 0.05.

^b^Cohen's kappa coefficient was used to analyse the consistency with high NLRP3 expression, *p* < 0.05.

^c^Cohen's kappa coefficient was used to analyse the consistency with MDSCs enrichment, *p* < 0.05.

### 2.2. Correlations between Fn infection, high NLRP3 expression and MDSCs enrichment and the clinicopathological features of ESCC patients

The chi-square test showed that Fn infection, high NLRP3 expression and MDSCs enrichment were related to sex, smoking status, drinking consumption, differentiation degree, invasion depth, lymph node metastasis and clinical stage but not to age (Table 3).

**Table 3.** Correlations between Fn infection, high NLRP3 expression and MDSCs enrichment and the clinicopathological characteristics of ESCC patients [*N* (%)].

Clinical features	*n*	Fn		*χ^2^*	*P*	NLRP3		*χ^2^*	*P*	MDSCs		*χ^2^*	*P*
		Positive	Negative			Positive	Negative			Positive	Negative		
Gender				24.91	<0.001			37.62	<0.001			25.12	<0.001
Male	160	71 (27.52)	89 (34.49)			97 (37.60)	63 (24.42)			67 (25.97)	93 (36.05)		
Female	98	14 (5.43)	84 (32.56)			21 (8.14)	77 (29.84)			12 (4.65)	86 (33.33)		
Age/years				0.69	0.406			2.29	0.130			1.10	0.294
≥60	140	43 (16.67)	97 (37.60)			58 (22.48)	82 (31.78)			39 (15.12)	101 (39.15)		
<60	118	42 (16.28)	76 (29.45)			60 (23.26)	58 (22.48)			40 (15.50)	78 (30.23)		
Smoking				58.33	<0.001			31.51	<0.001			56.43	<0.001
Positive	128	71 (27.52)	57 (22.09)			81 (31.40)	47 (18.22)			67 (25.97)	61 (23.64)		
Negative	130	14 (5.43)	116 (44.96)			37 (14.34)	93 (36.04)			12 (4.65)	118 (45.74)		
Alcohol				63.88	<0.001			36.90	<0.001			61.60	<0.001
Positive	124	71 (27.52)	53 (20.54)			81 (31.40)	43 (16.67)			67 (25.97)	57 (22.09)		
Negative	134	14 (5.43)	120 (46.51)			37 (14.33)	97 (37.60)			12 (4.65)	122 (47.29)		
Differentiation Type				45.13	<0.001			38.116	<0.001			52.60	<0.001
Poorly differentiated	51	37 (14.34)	14 (5.43)			43 (16.67)	8 (3.10)			37 (14.34)	14 (5.43)		
Moderately/Well differentiated	207	48 (18.60)	159 (61.63)			75 (29.07)	132 (51.16)			42 (16.28)	165 (63.95)		
Lymph node metastasis				88.45	<0.001			120.94	<0.001			86.86	<0.001
Positive	129	78 (30.23)	51 (19.77)			103 (39.92)	26 (10.08)			74 (28.68)	55 (21.32)		
Negative	129	7 (2.71)	122 (47.29)			15 (5.81)	114 (44.19)			5 (1.94)	124 (48.06)		
Depth of infiltration				35.74	<0.001			90.80	<0.001			30.73	<0.001
≥Adventitia	176	79 (30.62)	97 (37.60)			116 (44.96)	60 (23.26)			73 (28.29)	103 (39.92)		
<Adventitia	82	6 (2.33)	76 (29.45)			2 (0.78)	80 (31.00)			6 (2.33)	76 (29.46)		
Clinical stages				122.85	<0.001			173.82	<0.001			119.16	<0.001
I/II	147	7 (2.72)	140 (54.26)			15 (5.81)	132 (51.16)			5 (1.94)	142 (55.04)		
III/IV	111	78 (30.23)	33 (12.79)			103 (39.92)	8 (3.11)			74 (28.68)	37 (14.34)		

### 2.3. Correlations between Fn infection, high NLRP3 expression and MDSCs enrichment and the 5-year survival of ESCC patients

**Figure 3.**
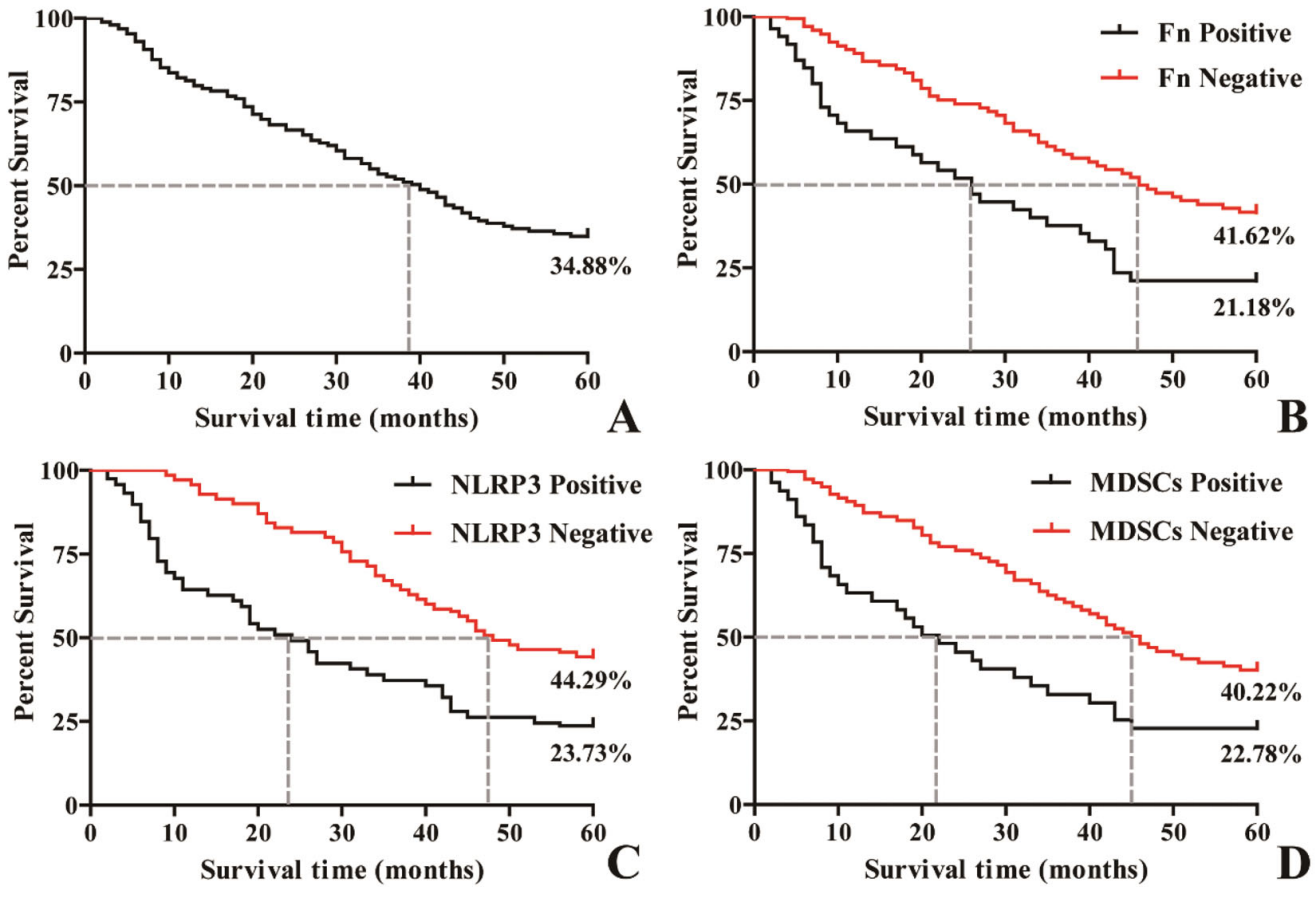
Kaplan–Meier survival curves of ESCC patients at 5 years after surgery. A: Five-year survival curve of patients with ESCC after surgery; B: Survival curves of patients with and without Fn infection; C: Survival curves of ESCC patients with and without NLRP3 expression; D: Survival curves of ESCC patients with and without MDSCs infiltration.

Kaplan–Meier survival analysis showed that the median and quartile of five-year survival and survival time among 258 ESCC patients were 34.88% and 40.00 (-∼19.00) months, respectively. The five-year survival rate and median and quartile of survival time in the positive group with Fn infection, high NLRP3 expression and MDSCs enrichment were significantly lower than those in the negative group (Figure 3 and Table 4, *p* < 0.05).

**Table 4.** Correlations of Fn infection, high NLRP3 expression and MDSCs enrichment with 5-year survival in ESCC patients.

	25%		50%		75%		$\chi^{2}$	*P*
	Est.	Std. Error	Est.	Std. Error	Est.	Std. Error		
Fn								
Positive	43.00	–	26.00	3.22	8.00	1.23	20.18	<0.001
Negative	–	–	46.00	3.95	24.00	2.89		
NLRP3								
Positive	53.00	–	24.00	2.44	8.00	0.81	25.81	<0.001
Negative	–	–	48.00	5.13	31.00	1.97		
MDSCs								
Positive	45.00	–	22.00	3.55	8.00	1.01	19.82	<0.001
Negative	–	–	46.00	3.01	26.00	2.90		
Overall	–	–	40.00	2.58	19.00	2.12		

### 2.4. Effects of Fn on the coculture of human PBMCs and ESCC cells

**Figure 4.**
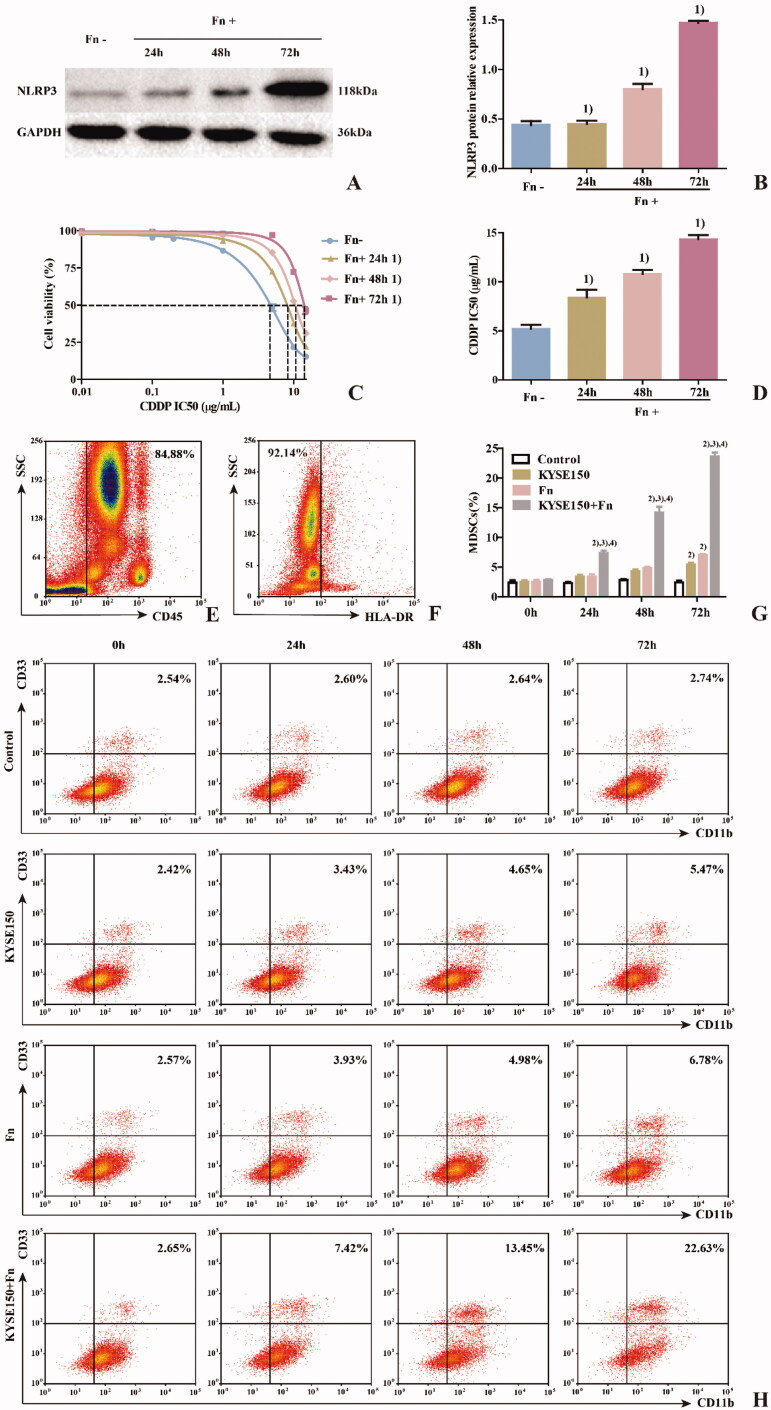
Effects of Fn on the coculture system of human PBMCs and ESCC cells. A: Western blot was used to detect the expression of NLRP3 protein in each cell line; B: Differences in NLRP3 protein expression among the groups of cell lines; C: Dose–response curve of CDDP; D: Differences in CDDP IC50 values among the groups of cell lines; E: Leukocyte population (CD45^+^ cells); F: HLA-DR^−^ Cell population; G: Differences in percentage of MDSCs (CD45^+^HLA-DR^−^CD11b^+^CD33^+^ cells) in each group; H: Flow cytometry of the percentage of MDSCs (CD45^+^HLA-DR^−^CD11b^+^CD33^+^ cells) in each group. 1) Compared with the Fn uninfected group, *p* < 0.05; 2) Compared with the control group, *p* < 0.05; 3) Compared with the KYSE150 group, *p* < 0.05; 4) Compared with the KYSE150 + Fn group, *p* < 0.05.

To detect the effect of Fn on MDSCs in the PBMC coculture system, PBMCs from healthy persons were cocultured with KYSE150 cells with or without Fn treatment and divided into four groups (Figure 4(H)) to simulate changes in the tumour microenvironment. The percentage of MDSCs among the PBMCs (Figure 4(H)) was detected by flow cytometry at different time points (24 h, 48 h and 72 h). The results (Figure 4(G)) showed that compared with the control group, the percentages of MDSCs in the KYSE150 group and Fn group increased at 72 h (*p* < 0.05). However, the percentage of MDSCs in the KYSE150 + Fn group increased at 24 h (*p* < 0.05). Meanwhile, compared with the KYSE150 and Fn groups, the KYSE150 + Fn group had a significantly increased percentage of MDSCs (*p* < 0.05). It was suggested that the percentage of MDSCs could be increased by coculturing PBMCs with tumour cells or by infection with Fn. The combination of these two regimens produced the most significant increase in the percentage of MDSCs in a time-dependent manner.

To detect the effect of Fn on the ESCC cell line KYSE150 in the coculture system, western blot and CCK-8 assays were used to detect the expression of NLRP3 and the IC50 value of CDDP in each cell line. The results showed that the expression levels of NLRP3 (Figure 4(A,B)) and the IC50 value of CDDP (Figure 4(C,D)) in the Fn-infected group were significantly higher than those in the Fn-uninfected group (*p* < 0.05). The two indexes in the infected group increased gradually with prolonged Fn infection time (*p* < 0.05). These results suggest that Fn infection can induce NLRP3 overexpression in KYSE150 cells in a coculture system and can induce resistance to CDDP treatment in a time-dependent manner.

### 2.5. Role of NLRP3 in Fn-induced CDDP resistance in ESCC cells

**Figure 5.**
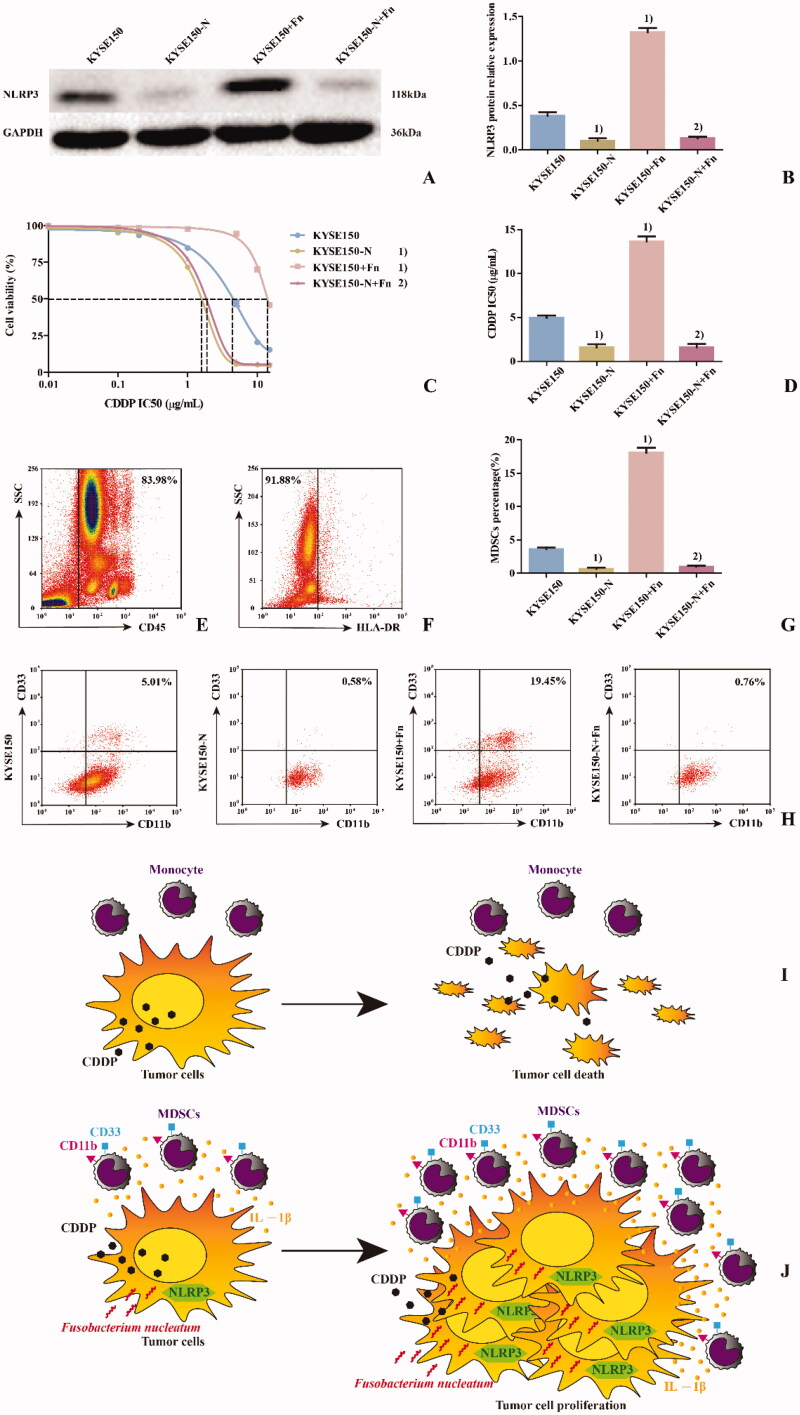
NLRP3 is a key regulatory factor of Fn-induced MDSCs enrichment and CDDP treatment resistance in ESCC. A: Western blot was used to detect the expression of NLRP3 protein in each cell line; B: Differences in NLRP3 protein expression among the groups of cell lines; C: Dose–response curve of CDDP; D: Differences in CDDP IC50 values among the groups of cell lines; E: Leukocyte population (CD45^+^ cells); F: HLA-DR^−^ Cell population; G: Differences in percentage of MDSCs (CD45^+^HLA-DR^−^CD11b^+^CD33^+^ cells) in each group; H: Flow cytometry of the percentage of MDSCs (CD45^+^HLA-DR^−^CD11b^+^CD33^+^ cells) in each group; I: Scientific schematic diagram of CDDP inducing the death of tumour cells not infected by Fn; J: Scientific schematic diagram of the enrichment of MDSCs caused by Fn via the activation of NLRP3 in ESCC cells and resistance to CDDP treatment. 1) Compared with the KYSE150 group, *p* < 0.05, and 2) compared with the KYSE150 + Fn group, *p* < 0.05.

To explore the potential molecular mechanism of Fn-induced CDDP resistance in ESCC, the NLRP3-knockdown KYSE150-N-cell line was established. PBMCs from healthy individuals were cocultured with KYSE150 or KYSE150-N cells and simultaneously infected or not infected with Fn. Then, they were divided into four groups (Figure 5(A)) to simulate tumour microenvironments. The percentage of MDSCs in each coculture system was detected by flow cytometry. The results (Figure 5(G,H)) showed that compared with the KYSE150 coculture system, the percentage of MDSCs in the KYSE150 + Fn coculture system was significantly increased (*p* < 0.05), while the percentage of MDSCs in the KYSE150-N coculture system was significantly decreased (*p* < 0.05). However, compared with the KYSE150-N coculture system, there was no significant difference in the percentage of MDSCs in the KYSE150-N + Fn coculture system. These results suggest that NLRP3 is a key regulator of MDSCs enrichment induced by Fn infection. The viability of CDDP treatment in each cell line was detected by the CCK8 method. The results (Figure 5(C,D)) showed that compared with KYSE150 cells, KYSE150 + Fn cells had a significantly higher CDDP IC50 value (*p* < 0.05), while that of KYSE150-N cells was significantly decreased (*p* < 0.05). However, compared with the KYSE150-N cells, there was no significant difference in the CDDP IC50 value in KYSE150-N + Fn cells. These results suggest that NLRP3 is a key regulatory factor of Fn-induced resistance to CDDP.

### 2.6. Effects of Fn infection and NLRP3 knockdown on CDDP treatment of ESCC detected in NSG mice

**Figure 6.**
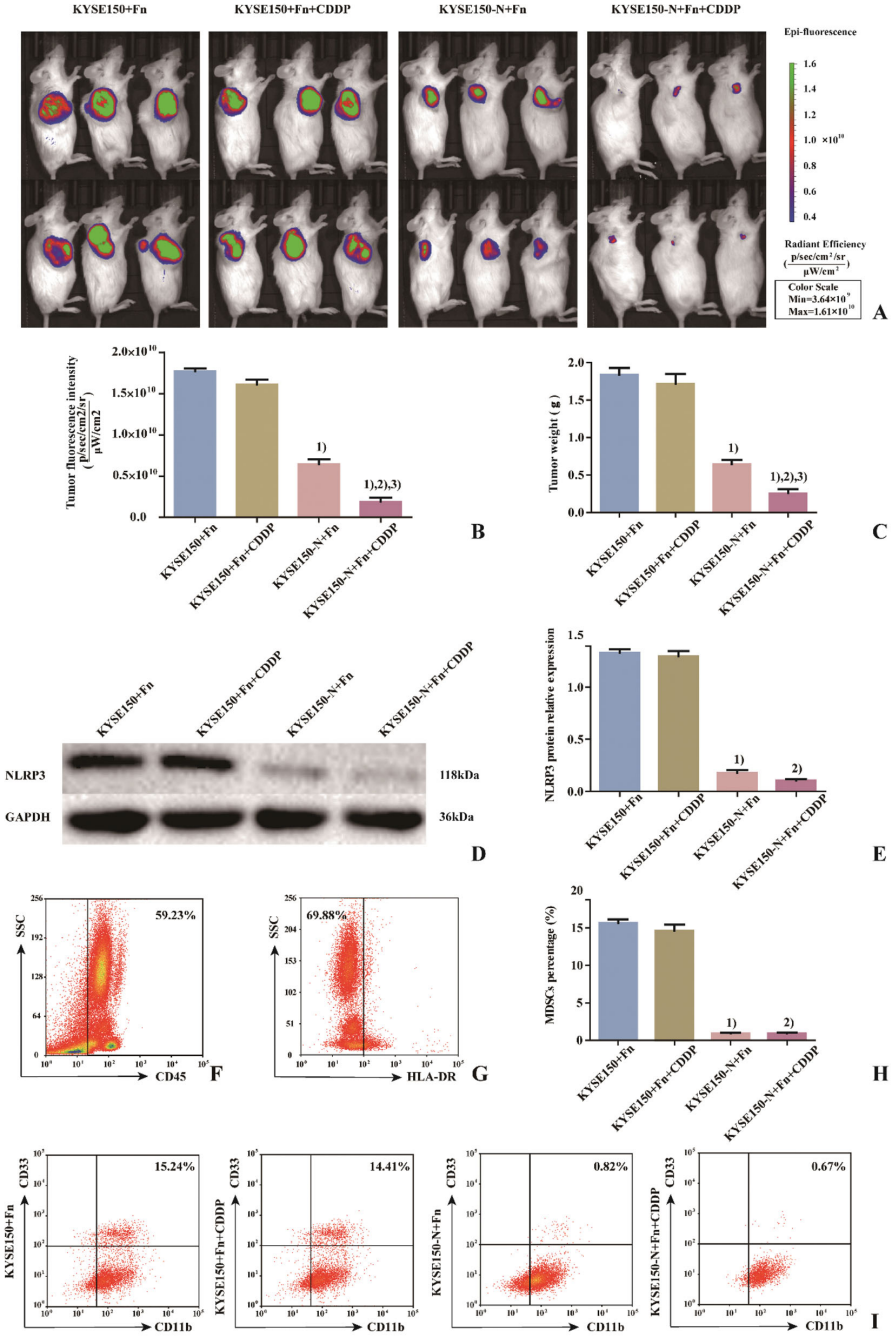
Effects of Fn infection and NLRP3 knockdown on CDDP treatment in NSG mice. A: Tumour size and fluorescence intensity of lesions from NSG mice in each group detected by in vivo imaging; B: Differences in tumour size and fluorescence intensity of NSG mice among the groups; C: Differences in tumour weight of NSG mice among the groups; D: Western blot was used to detect the expression of NLRP3 protein in tumours of NSG mice among the groups; E: Differences in NLRP3 protein expression in tumours of NSG mice among the groups; F: Leukocyte population (CD45^+^ cells); G: HLA-DR^−^ Cell population; H: Differences in percentage of MDSCs (CD45^+^HLA-DR^−^CD11b^+^CD33^+^ cells) in tumours of NSG mice among the groups; I: Flow cytometry of the percentage of MDSCs (CD45^+^HLA-DR^−^CD11b^+^CD33^+^ cells) in tumours of NSG mice among the groups. 1) Compared with the KYSE150 + Fn group, *p* < 0.05; 2) compared with the KYSE150 + Fn + CDDP group, *p* < 0.05; 3) compared with the KYSE150-N + Fn group, *p* < 0.05.

To detect the effects of Fn infection and NLRP3 knockdown on CDDP treatment of ESCC, NSG mice were divided into 4 groups (Figure 6(A)). KYSE150 + Fn cells and KYSE150-N + Fn cells were subcutaneously inoculated into the NSG mice, and human PBMCs were injected into the caudal vein to establish an *in vivo* animal model to simulate the tumour microenvironment. The *in vivo* imaging results (Figure 6(A,B)) and tumour weight detection results (Figure 6(C)) showed that ① Compared with the KYSE150 + Fn group, there was no significant difference in the tumour-forming ability of NSG mice in the KYSE150 + Fn + CDDP group, suggesting that the tumours in this group were no longer sensitive to CDDP treatment. ② Compared with the KYSE150 + Fn and KYSE150 + Fn + CDDP groups, NSG mice in the KYSE150-N + Fn and KYSE150-N + Fn + CDDP groups had significantly reduced tumour-forming ability (*p* < 0.05), suggesting that NLRP3 knockdown could significantly inhibit the malignant proliferation of tumours. ③ Compared with the KYSE150-N + Fn group, NSG mice in the KYSE150-N + Fn + CDDP group showed a significantly reduced tumour-forming ability (*p* < 0.05), suggesting that NLRP3 knockdown combined with CDDP treatment could more effectively inhibit malignant proliferation. Subcutaneous tumours from NSG mice in each group were excised and processed. After grinding the samples, the specimen from each NSG mouse was divided into two parts: one part was used for protein extraction for the detection of NLRP3 protein expression in NSG mice in each group by western blot (Figure 6(D,E)), and the other part was prepared as a single-cell suspension for the detection of the percentage of MDSCs in NSG mice in each group by flow cytometry (Figure 6(H,I)). The results showed that compared with the KYSE150 + Fn and KYSE150 + Fn + CDDP groups, the percentage of MDSCs in NSG mice in the KYSE150-N + Fn and KYSE150-N + Fn + CDDP groups was significantly decreased by NLRP3 knockdown (*p* < 0.05), suggesting that the enrichment of MDSCs was closely related to NLRP3 expression.

## 3. Discussion

The prognosis of ESCC is very poor because early diagnosis is difficult, and the five-year survival rate of patients with intermediate- and advanced-stage disease is extremely low [27]. Chemotherapy is the main treatment for patients with advanced ESCC, and CDDP is one of the most powerful and widely used chemotherapy drugs. In tumour cells, CDDP can interact with DNA to form a DNA-crosslinking agent to induce cytotoxicity; activate Erk, p53, p73, MAPK and other signal transduction pathways; and promote tumour cell death. However, the development of drug resistance seriously interferes with its clinical efficacy [28–29]. Therefore, it is of great significance to identify the molecular mechanism of CDDP resistance in ESCC to improve treatment methods and survival rates. Data have shown that pathogenic microbial infection can reduce the response of ESCC to conventional chemotherapy drugs [30]. In this study, it was found that the sensitivity of ESCC to CDDP gradually decreased with prolonged Fn infection time, suggesting that Fn infection can induce resistance to CDDP treatment in ESCC, but the specific pathogenic mechanism remains unclear.

Data [31] show that the host’s first protective barrier against invasion by pathogenic microorganisms is the body’s innate immune system, which is mainly composed of inflammatory complexes. NLRP3, as an important member of the inflammatory complex family, monitors danger signals in the cytoplasm, identifies various pathogenic microorganisms and their metabolites, regulates the immune response, causes inflammatory responses [32], enhances the proliferation, invasion and anti-apoptotic ability of tumour cells, and reduces the sensitivity of tumour cells to chemotherapy drugs [33]. *Helicobacter pylori* [34], Epstein Barr virus [35] and HPV [36] can induce high expression of NLRP3 in tumour cells; promote malignant proliferation, distant metastasis, resistance to apoptosis and angiogenesis; and significantly reduce the efficacy of chemotherapy in patients.

Studies have shown that MDSCs are important immunosuppressive cells in the tumour microenvironment. Melanoma [37], prostate [38] and kidney cancer [39] patients with low levels of MDSCs responded well to conventional chemotherapy, while patients with high levels of MDSCs were insensitive to chemotherapy and prone to distant metastasis. These results suggest that MDSCs are the main obstacle to successful tumour immunotherapy and one of the important reasons for resistance to multiple drug therapies. Clinical data show that a variety of pathogenic microorganisms can enrich MDSCs, regulate intercellular inhibition mechanisms, secrete immunosuppressive molecules, reshape the tumour microenvironment, weaken the immune response in the body, and help tumour cells escape immune surveillance. Both hepatitis B virus and *Helicobacter pylori* can induce the enrichment of MDSCs in cancer tissues, resulting in an imbalance in the proportion of CD4^+^ and CD8^+^ T-cell subsets and producing the inactivation of antitumour immunity and the malignant proliferation of cancer cells [40–41]. Additionally, the activation of NLRP3 can reshape the tumour immune microenvironment, recruit MDSCs, weaken the body's antitumour immunity, assist tumour cells in resisting chemotherapy, and reduce the sensitivity of tumour cells to oxaliplatin [42]. The percentage of MDSCs in melanoma, thymoma, and breast cancer in mice was decreased with NLRP3 deletion, but gemcitabine and 5-FU showed significantly increased antitumour efficacy [33,43] and significantly prolonged survival.

This study found a high degree of consistency between Fn infection, high NLRP3 expression and MDSCs enrichment in the cancer tissues from 258 ESCC patients treated with conventional TP chemotherapy. These results suggested that NLRP3 was highly activated in FN-infected ESCC cells and that MDSCs were enriched in the microenvironment. Patients in the positive group were mostly male smokers and drinkers, indicating that long-term smoking and drinking led to a poor oral environment. Additionally, they were more prone to Fn infection and colonisation, the NLRP3 colonisation site was mostly highly expressed in these patients, and a large amount of MDSCs infiltration was present. The positive rate of these three characteristics in ESCC with low differentiation was higher than that of ESCC with medium–high differentiation, suggesting that these three characteristics were related to the degree of malignancy. Meanwhile, in the positive group of ESCC patients, lymph node metastasis occurred early, infiltration was deep, and the clinical stage occurred late, suggesting that Fn infection, high NLRP3 expression, and MDSCs enrichment may promote the malignant progression of ESCC. The five-year survival rate and median survival time of patients in the positive group were significantly lower than those in the negative group, suggesting that the effective elimination of Fn and the inhibition of high NLRP3 expression and MDSCs enrichment prolongs the survival of ESCC patients undergoing conventional postoperative TP chemotherapy.

To explore the potential molecular mechanism of Fn-induced CDDP resistance in ESCC, this study established a coculture system of human peripheral blood PBMCs and ESCC cells to simulate the tumour immune microenvironment. Fn could induce an increase in the percentage of MDSCs in the coculture system and could also induce high expression of NLRP3 and resistance to CDDP treatment in ESCC cells. This result suggested that the Fn-induced activation of NLRP3 and remodelling of the immune microenvironment weakened the sensitivity of ESCC cells to CDDP. This study also found that when NLRP3 knockdown was observed in ESCC cells, the percentage of MDSCs in the coculture system and the CDDP IC50 value were significantly decreased. However, Fn infection of NLRP3-knockdown ESCC cells and the PBMC coculture system did not increase either of these parameters. These results suggest that NLRP3 is a key regulator of MDSCs enrichment and CDDP resistance induced by Fn infection. In the subcutaneous tumour-bearing experiment involving NSG mice, due to the high expression of NLRP3 induced by Fn infection, a large number of MDSCs were enriched in the tumour and were no longer sensitive to CDDP treatment. Knockdown of NLRP3 can inhibit MDSCs and effectively control malignant tumour proliferation. These results indicate that Fn can induce the high expression of NLRP3 in ESCC cells, lead to the enrichment of MDSCs, cause immune inactivation, lead to CDDP treatment resistance, and promote the malignant proliferation of cancer cells.

In summary, since the occurrence and development of tumours is a multifactor and multistep evolution process, the specific pathogenic mechanism of Fn needs to be further explored. However, disrupting the permanent colonisation of Fn in the host and effectively inhibiting NLRP3 expression and MDSCs enrichment are of great significance for actively and effectively improving the clinical efficacy of CDDP and prolonging the survival of patients with ESCC.

Author contributions

Fuyou Zhou: Conceptualization, Supervision;

Mengxia Liang: Writing-Reviewing and Editing;

Yiwen Liu: Writing-Original draft preparation;

Mengxia Liang, Zheyuan Zhang, Haijun Yang and Ningtao Dai: Methodology, Investigation;

Jinyu Kong, Wei Sun: Software;

Haijun Yang, Ningtao Dai and Mengxia Liang: Investigation, Data curation;

Ning Zhang, Xiaopeng Wang, Yibo Guo and Min Wang: Validation, Visualization.

## Data Availability

The data that support the findings of this study are available on request from the corresponding author. The data are not publicly available due to privacy or ethical restrictions.
